# The benefits of leisure activities on healthy life expectancy for older people with diabetes

**DOI:** 10.1186/s13098-024-01347-3

**Published:** 2024-05-14

**Authors:** I-Wen Huang, Shao-Jen Weng, Cheng-Hsi Liao, Yeong-Yuh Xu, Liang-Po Hsieh, Shih-Chia Liu, Yao-Te Tsai

**Affiliations:** 1 Department of Endocrinology and Metabolism, Chung-Kang Branch, Cheng Ching Hospital, Taichung, 407 Taiwan; 2 Department of Industrial Engineering and Enterprise Information, Tunghai University, Taichung, 407 Taiwan; 3 Department of Surgery, Taichung Armed Forces General Hospital, Taichung, 411 Taiwan; 4 National Defense Medical Center, Taipei, 114 Taiwan; 5 Department of Artificial Intelligence and Computer Engineering, National Chin-Yi University of Technology, Taichung, 411 Taiwan; 6 Department of Neurology, Chung-Kang Branch, Cheng Ching Hospital, Taichung, 407 Taiwan; 7 Department of Nursing, Hungkuang University, Taichung, 433 Taiwan; 8 Department of Information Management, National Kaohsiung University of Science and Technology, Kaohsiung, 824 Taiwan

**Keywords:** Leisure activities, Diabetes mellitus, Life expectancy, Healthy life expectancy

## Abstract

**Purpose:**

The purpose of this study is to examine whether leisure activities can help reduce years lived with disability and increase healthy life expectancy of diabetics aged 50 years and above.

**Methods:**

Analysis was based on five waves of follow-up survey data (Taiwan Longitudinal Study of Aging, TLSA) from 1996 to 2011. A total of 5131 participants aged 50 years and above in 1996 were included in the analysis, and gender, leisure activity participation, and diabetes mellitus were used as primary variables to examine the variation trend in health status in the participants. The health status in the various waves of surveys was measured using the activities of daily living scale, and nondisabled was defined as healthy. A multivariate logistic regression model was used to calculate the life expectancy (LE) and healthy life expectancy (HLE) of the people aged 50 years and above.

**Results:**

The diabetes older people with a high frequency of leisure activities have longer HLE than those with lower activity frequency. Using 50-year-old diabetic women as an example, the LE (HLE) of those with six or more leisure activities and those with three or fewer leisure activities was 30.40 (25.34) and 24.90 (20.87), respectively. The LE (HLE) of men with the same conditions was 24.79 (22.68) and 20.30 (18.45), respectively.

**Conclusions:**

This study used life expectancy and healthy life expectancy as markers to evaluate health benefits and provided evidence that leisure activities can help extend the life span and maintain the health status of middle-aged and older diabetics.

## Introduction

With the dawn of an aged society, Taiwan’s demographic structure has changed to one with low birth and mortality rates. Due to the effects of the low mortality rate, the life expectancy in Taiwan has increased, of which the increase in older people aged 65 years and above is more significant than in other age groups. According to the Republic of China Population Estimate (2022 to 2070) [1], Taiwan will become a super-aged society in 2025. In fact, Taiwan’s 65 years and above population officially transitioned into the older people’s demographic in late May 2018. The high proportion of older people has resulted in huge care and medical expenditure pressure on the Taiwanese society.

Population aging and an increase in life expectancy do not necessarily imply an improvement in the quality of life. The rise in the physically impaired population or person-years leads to considerable medical and long-term care expenses. Conversely, chronic disease is one of the leading causes of older people’s deaths globally and includes malignancies, heart disease, cerebrovascular disease, diabetes, and hypertension. Due to reduced metabolism, middle-aged and older people are susceptible to chronic disease and its complications, which increases the risk of death. It can be seen that the healthcare burden brought about by chronic disease will accompany the aged society of Taiwan. Besides affecting the physical and mental status of individuals, chronic diseases can also increase family expenditure due to healthcare, of which diabetes mellitus is common in older people.

Among the top 10 causes of death in Taiwan, cerebrovascular disease, cardiovascular disease, and nephropathy have an inseparable causal relationship with poor diabetes control. Hence, it can be seen that diabetes has a huge impact on Taiwanese. According to the National Health Insurance Administration statistics, around 2.25 million insured people sought treatment for diabetes in 2020, of which the age group with the highest number of patients was 1.13 million, which was twice the average. When viewed from the perspective of health insurance fees, the health insurance fee points per capita for diabetes in 2020 was around 11,818 points [2]. Although diabetes is not the disease with the highest medical expenditure, the costs are considerable when the points for related complications are added.

The life satisfaction of older people will decrease when they suffer from chronic diseases [3]. Overseas studies also showed that health status not only reflects an individual’s actual health but also their subjective thoughts and has direct effects on life satisfaction [4–6]. Frequent interactions with friends and family and moderate participation in social activities, socializing, and community activities will increase the life satisfaction of older people [3, 7–11]. A study also pointed out that family-based leisure services and social interaction opportunities can maintain the health status of older people and promote physical activities and social welfare in older people, particularly during the COVID-19 pandemic [12]. Leisure activities not only result in physical relaxation, promote physical and mental health, and increase physical strength but also help relieve emotional stress and improve the quality of life in older people [5, 13–16].

Recently, many studies have examined the relationship between leisure activities and health in older people. Leisure activities, such as traveling, can improve the health status of older people [17]. Another study examined the effect of different leisure activities (active or passive) on healthy aging and happiness and found that passive leisure activities may impede a socially healthy aging process in older people [18]. Col et al. [19] pointed out that leisure activities have significant effects on happiness, life satisfaction, depression, and stress levels in older people but do not have significant effects on the levels of anxiety and mental health. Kim et al. [20] used a questionnaire survey to examine the welfare development measures for older people and pointed out that the quality of life, subjective health status, and family income of older people are related to participation in leisure activities. Hakman et al. [21] provided a theoretical basis for the planning and management of older people’s leisure and health promotion activities. They pointed out that the planning and implementation of leisure and health activity plans can improve the physical working capacity, psycho-emotional state, the level of pain, cognitive functions, and the level of somatic health of older people. The study by Jeong and Park [22] showed that leisure participation and exploration are significantly correlated with depression and quality of life in older people. The study by Sala et al. [23] found that participation in leisure activities is extremely beneficial to older people as it can help them maintain healthy cognitive, physical, and mental health during aging. Zhou et al. [24] examined the relationship between leisure activities and frailty in older people and pointed out that leisure activities can decrease the risk of frailty. In addition, many studies also pointed out that leisure activity participation has many benefits for older people, such as promoting mental health [25, 26], health promotion [27, 28], improving family relationships and interpersonal interactions [29–32], and increasing financial benefits [32, 33].

In summary, in view of the huge impact of leisure activities on older people health, particularly diabetics, the primary objective of this study was to understand whether leisure activity participation by people aged 50 years and above can increase the health benefits in diabetics. The average life expectancy and the gap in healthy life expectancy were compared based on the diabetes situation of older Taiwanese people and their participation in leisure activities to examine the health benefits of leisure activities for older people with diabetes.

## Materials and methods

### Data source

This study is a long-term cohort study that is representative of Taiwan. The data used in this study was obtained from the Taiwan Longitudinal Study of Aging (TLSA) by the Health Promotion Administration, Ministry of Health. Data from the 3rd to 7th surveys in 1996 to 2011 (1996, 1999, 2003, 2007, 2011) were used for analysis, and the 3rd older people survey in 1996 was used as the starting year for data analysis. People aged 50 years and above in the 1996 survey were included in this study as participants, and there were 5131 participants in total. Besides collecting data using past questionnaire surveys, the sample’s identification card number and cause of death data were compared, and the sample survival status and date of death were updated year by year. The Taiwan Longitudinal Study of Aging (TLSA) database will be used to examine the correlation between leisure activities and healthy life expectancy in this study.

### Definition and measurement of variables

This study used leisure activity participation in the starting year as the primary variable. The health and survival statuses observed during the 15-year follow-up survey period from 1996 to 2011 were used to calculate life and healthy life expectancies. In order to control the effects of basic demographic characteristics on health, a statistical model was used to control the effects of age and gender. The definitions of basic demographic characteristics, disease, leisure activities, and health status of the participants are as follows:

1. Basic demographic characteristics: age, gender (male, female).
2. Disease definition: Since many comorbidities, such as cerebral vascular diseases, cardiovascular disorders, and nephropathy, were closely related to diabetes mellitus, this study focuses on the occurrence of diabetes. The diagnosis of diabetes was confirmed by the physician (yes, no) during the follow-up survey.
3. Definition of leisure activities: The frequency of leisure activity participation was used as the main variable for analysis and examination. The leisure activities include:

- Watching television/videotapes.
- Listening to the radio.
- Reading newspapers, magazines, books, or novels.
- Reciting sutras, burning incense, praying or reading sutras in the temple, praying, going to church (religious activities).
- Playing chess or cards (including mahjong, four color cards).
- Chatting or drinking tea with relatives, friends, or neighbors.
- Gardening and horticulture.
- Taking a walk.
- Slow job, hiking, playing ball games, and other outdoor activities.
- Participation in team activities, such as concerts, dancing, and Tai chi.
- Interests or hobbies: playing a musical instrument, painting, woodworking, needlework, stamp collecting, collection, etc.
- Watching concerts, Taiwanese operas, and Peking operas.

4. Definition of health status: In this study, healthy life expectancy was defined as disability-free life expectancy and health status was measured using the activities of daily living (ADL) scale, that is, Katz Index [34, 35]. This index was based on the first six items in the Barthel scale and includes the following six activities. Disability is when a participant finds it “somewhat difficult”, “difficult,” or “completely impossible” to complete any of the following activities.

- Bathing.
- Dressing/undressing.
- Feeding.
- Getting out of bed, standing, or sitting on a chair.
- Walking indoors.
- Toileting.

### Statistical analysis

The discrete-time Markov model [36] was used as the analysis model for healthy life expectancy calculations in this study, and the maximum likelihood method was used to estimate transition probabilities for health states to calculate life expectancy [37]. The advantage of this method is that it can fully use all data of the participant, including the status of the participant in each wave of survey, survey date, and can also handle the missing values for lost-to-follow-up, health status, and examination data and is suitable for follow-up surveys with different intervals. The aforementioned 1996–2011 elderly disability health information was used to calculate the effects of leisure activity participation and diabetes on the healthy life expectancy of middle-aged and older people. At the same time, gender differences were included in the examination.

## Results

Table 1 shows the age, gender, diabetes status, duration, and disability status distribution of the 5131 study participants in the 1996 survey. From the leisure activity participation of people aged 50 years and above in this study, it can be seen that the mean age of those who participated in more leisure activities was lower (64.79). Among the 5131 participants, 559 were older people with diabetes in the 1996 survey. At the same time, it was found that there were more male participants (66.36%). In addition, diabetes and ADL disability examination found that diabetes decreased as the number of leisure activities increased (from 11.94 to 9.79%), and a similar trend was observed for disability (from 24.66 to 3.29%).

**Table 1:** Age, gender, diabetes status, and disability status distribution of samples aged 50 years and above in 1996 based on leisure activity participation

	Leisure activity participation		
	3 or less	4–5	6 or more types
**Variable**	n (%)	n (%)	n (%)
Age (mean ± SD)	67.79 ± 9.95	66.91 ± 9.04	64.79 ± 8.65
Gender: Male	854 (44.16)	1036 (54.90)	868 (66.36)
Diabetes: Yes	231 (11.94)	200 (10.60)	128 (9.79)
ADL disability: Yes	477 (24.66)	151 (8.00)	43 (3.29)

For analyzing the types of leisure activities, Table 2 shows the age, gender, and leisure activity participation of samples by the status of diabetes, disease duration (years: ≤5 and > 5), and ADL disability. From Table 2, watching TV (94.07%), drinking tea with friends or neighbors (60.52%), religious activities (58.58%), and taking a walk (56.32%) were highly participatory leisure activities. On the other hand, considering the duration of diabetes, results from Table 2 also show that whether the duration of diabetes is long or short, the four leisure activities (watching TV, drinking tea with friends or neighbors, religious activities, and taking a walk) are still the main leisure activities for the older people without ADL disability. For example, the proportions of watching TV, drinking tea with friends or neighbors, religious activities, and taking a walk for older people with a lower duration of diabetes (≤ 5 years) and without ADL disability were 97.97%, 60.16%, 64.49%, and 57.55%, respectively. For the case with longer duration of diabetes (> 5 years), these four proportions were 97.78%, 62.22%, 61.67%, and 74.42%, respectively.

**Table 2:** Age, gender, and types of leisure activity participation of samples aged 50 years and above in 1996 by the status of diabetes, disease duration (years: ≤5 and > 5), and ADL disability

		Diabetes					
		No		Yes			
				Duration (Years)			
				≤ 5		> 5	
		ADL disability					
	Total	No	Yes	No	Yes	No	Yes
Variable	n (%)	n (%)	n (%)	n (%)	n (%)	n (%)	n (%)
Age (mean ± SD)	66.70 ± 9.37	65.58 ± 9.14	74.23 ± 8.62	64.89 ± 8.23	71.96 ± 7.56	67.34 ± 7.21	72.46 ± 6.94
Male	2757 (53.80)	2267 (56.30)	226 (41.77)	119 (48.37)	24 (52.17)	85 (47.22)	35 (41.67)
The types of leisure activities							
Watching television/videotapes	4819 (94.07)	3872 (96.20)	432 (79.85)	241 (97.97)	37 (80.43)	176 (97.78)	60 (71.43)
Listening to the radio	1886 (37.00)	1553 (38.83)	149 (27.54)	86 (34.96)	10 (21.74)	65 (36.11)	22 (26.19)
Reading newspapers, magazines, books, or novels	1999 (39.14)	1733 (43.17)	82 (15.21)	86 (35.10)	10 (21.74)	72 (40.22)	16 (19.28)
Religious activities	3000 (58.58)	2478 (61.60)	217 (40.11)	148 (60.16)	20 (43.48)	112 (62.22)	24 (28.57)
Playing chess or cards	502 (9.83)	426 (10.62)	17 (3.14)	25 (10.20)	2 (4.35)	27 (15.08)	5 (6.02)
Chatting or drinking tea with relatives, friends, or neighbors	3096 (60.52)	2530 (62.94)	238 (43.99)	158 (64.49)	21 (46.67)	111 (61.67)	37 (44.05)
Gardening and horticulture	1527 (29.84)	1311 (32.61)	65 (12.01)	88 (35.77)	0 (0.00)	53 (29.44)	10 (12.05)
Taking a walk	2874 (56.32)	2375 (59.26)	180 (33.27)	141 (57.55)	15 (32.61)	133 (74.42)	29 (34.52)
Slow job, hiking, playing ball games, and other outdoor activities	943 (18.43)	856 (21.29)	8 (1.48)	39 (15.85)	1 (2.17)	35 (19.55)	4 (4.76)
Participation in team activities	410 (8.00)	347 (8.62)	11 (2.03)	24 (9.76)	0 (0.00)	24 (13.33)	4 (4.76)
Interests or hobbies	272 (5.31)	248 (6.16)	5 (0.92)	8 (3.25)	0 (0.00)	11 (6.11)	0 (0.00)
Watching concerts, Taiwanese operas, and Peking operas	259 (5.06)	224 (5.57)	10 (1.85)	11 (4.47)	1 (2.17)	11 (6.15)	2 (2.38)
Others	222 (4.33)	190 (4.72)	7 (1.29)	14 (5.69)	1 (2.17)	8 (4.44)	2 (2.38)

**Fig. 1:**
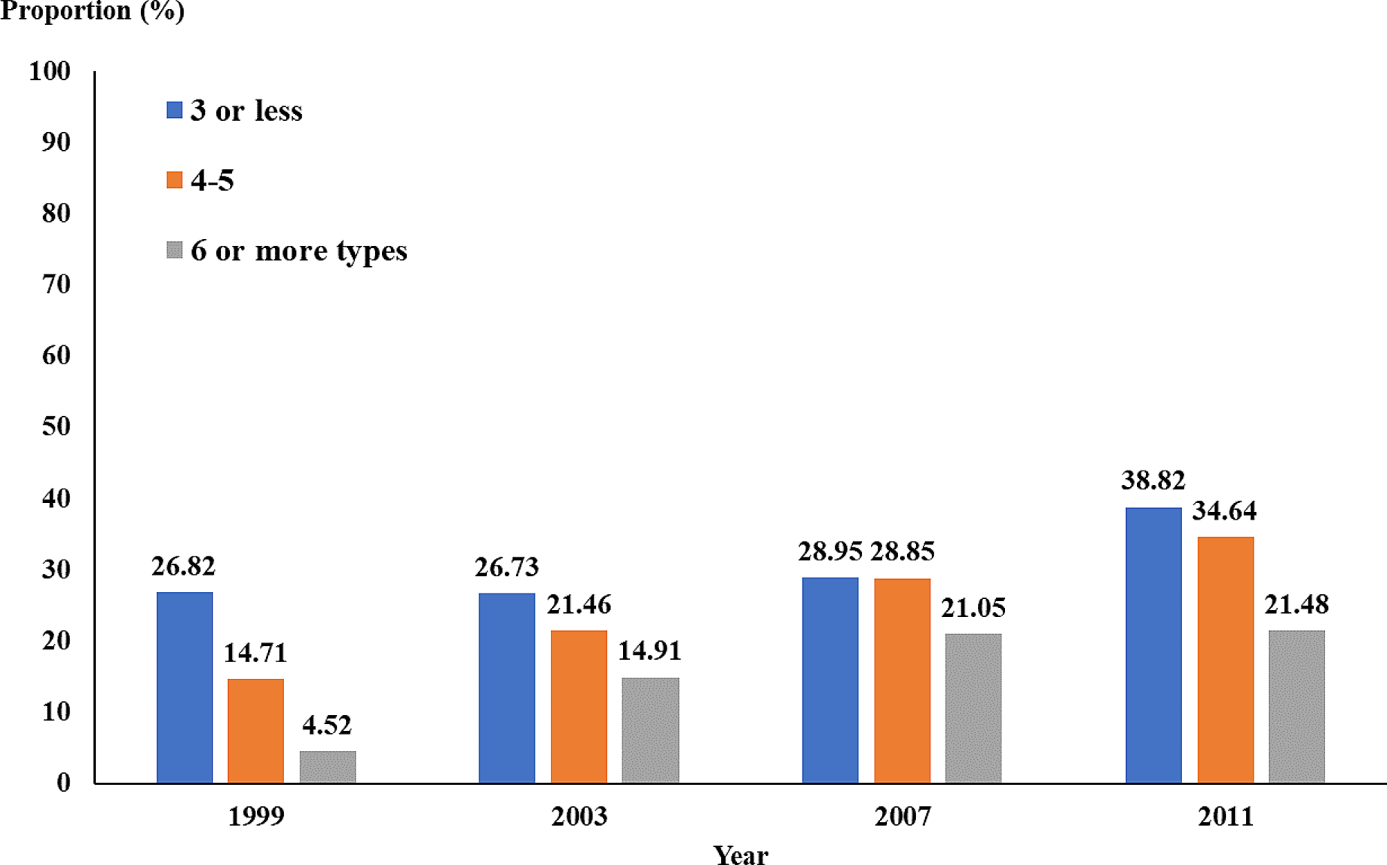
The proportion of diabetics with ADL disability based on leisure activity participation and year

Conversely, an examination of diabetics found that disability in diabetics increased with the year, that is, the proportion of people with disability increases with age. When classified based on leisure activity participation, the proportion of middle-aged and older diabetics with disability in the different years decreases with increasing leisure activity participation: the proportion of subjects with a disability who participated in 3 or fewer activities was 26.73-38.82%, the proportion of subjects with a disability who participated in 4–5 activities was 14.71–34.64%. In comparison, the proportion of dis-abled participants who participated in 6 or more activities was 4.52–21.48% (Fig. 1).

In addition, this study estimated the life expectancy and healthy life expectancy at 50, 60, 65, 75, and 85 years based on the diabetes status and leisure activity participation of middle-aged and older participants aged 50 years and above, which is shown in Table 3. Using 50 years as an example, the life expectancy (healthy life expectancy) of nondiabetics who participated in 3 or fewer, 4–5, and 6 leisure activities was 29.90 years (26.76 years), 32.03 years (28.83 years), and 33.76 years (30.72 years), respectively while the corresponding (healthy life expectancy) of diabetics was 22.97 years (19.91 years), 24.98 years (21.86 years), and 26.63 years (23.66 years), respectively. It can be seen that the life expectancy and healthy life expectancy of nondiabetics were longer than diabetics. Contrarily, increasing leisure activity participation also led to an increase in life expectancy and healthy life expectancy when the age is the same.

**Table 3:** Life expectancy and healthy life expectancy of middle-aged and older people based on age, diabetes status, and leisure activity participation

	Diabetes	No			Yes		
Age	Leisure activity participation	3 or less	4–5	6 or more	3 or less	4–5	6 or more
50	LE	29.90	32.03	33.76	22.97	24.98	26.63
	HLE	26.76	28.83	30.72	19.91	21.86	23.66
60	LE	21.19	23.15	24.72	15.30	17.01	18.39
	HLE	18.01	19.90	21.64	12.15	13.79	15.34
65	LE	17.33	19.14	20.58	12.20	13.70	14.88
	HLE	14.10	15.84	17.46	8.95	10.37	11.74
75	LE	11.13	12.52	13.56	7.88	8.93	9.61
	HLE	7.70	9.00	10.26	4.22	5.14	6.07
85	LE	7.43	8.41	8.93	6.00	6.82	7.02
	HLE	3.50	4.30	5.11	1.57	2.01	2.52

LE = life expectancy; HLE = Healthy life expectancy

**Fig. 2:**
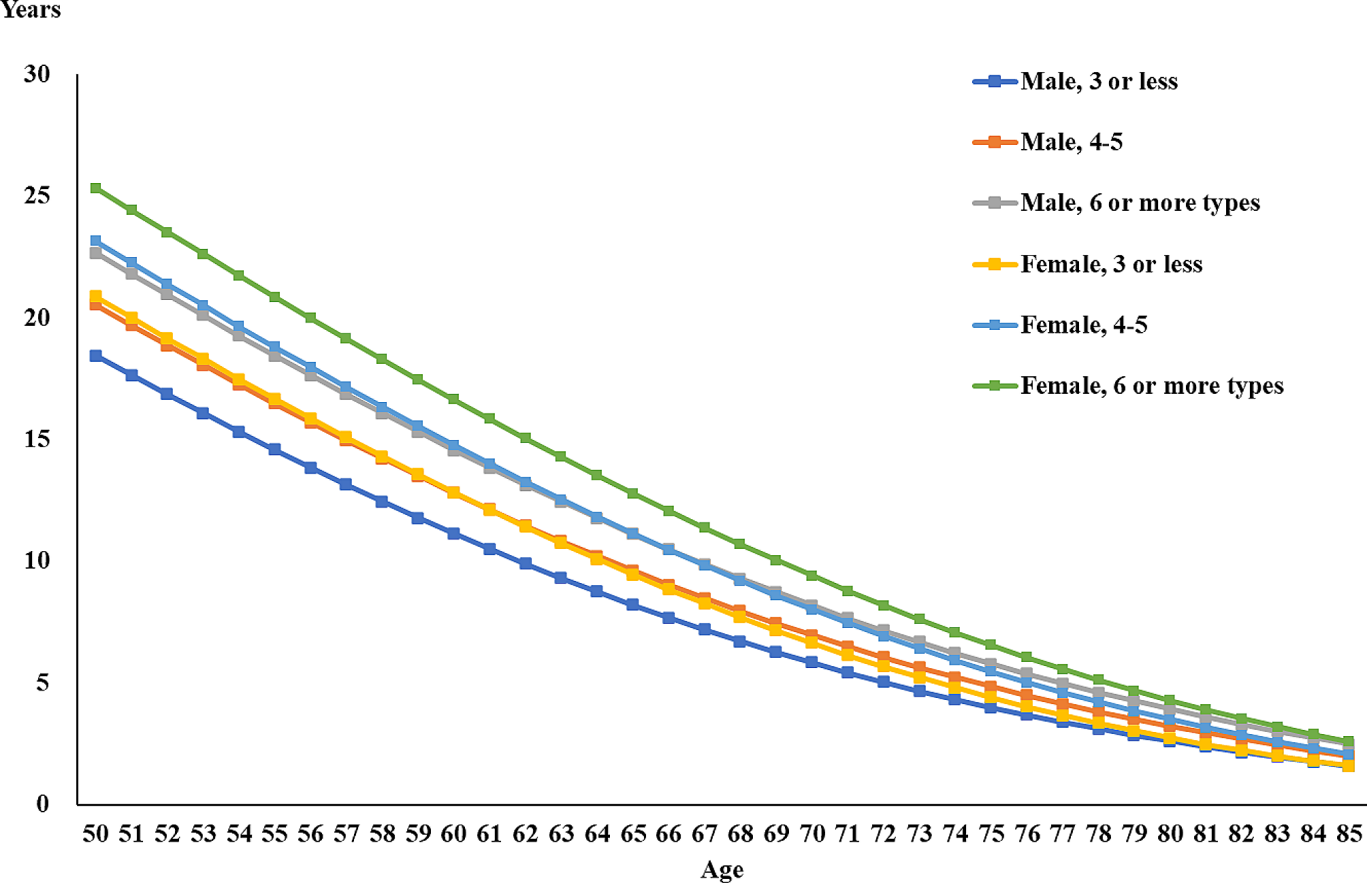
Healthy life expectancy trends of diabetics based on gender, leisure activity participation, and age

Figure 2 shows the healthy life expectancy trends of diabetics based on gender. It can be seen that the healthy life expectancy of females is longer than males under the same conditions, such as the same leisure activity participation and age. In addition, participation in more leisure activities can increase healthy life expectancy. For example, the healthy life expectancy of 50-year-old male diabetics who participated in 3 or fewer, 4–5, and 6 and more leisure activities was 18.45 years, 20.54 years, and 22.68 years, respectively, and the corresponding healthy life expectancy for females was 20.87, 23.17, and 25.34 years, respectively. The increase in healthy life expectancy due to increased leisure activities in both genders is around 2 years.

## Discussion and conclusions

Participation in leisure activities has many benefits for middle-aged and older diabetics, including physical health, mental health, and socializing. Moderate leisure activities such as walking, swimming, or yoga can help increase insulin sensitivity and promote fasting blood glucose and glycated hemoglobin level control. At the same time, these leisure activities can prevent cardiovascular disease risk in middle-aged and older diabetics. Some leisure activities, such as participation in cooking courses or health seminars, can provide practical diet and lifestyle management information to middle-aged and older people. This can help patients better understand how to manage diabetes and achieve better control. Furthermore, diabetics can decrease the risk of some diabetes-related complications, such as neuropathy or retinopathy, through participation in leisure activities.

On the other hand, results of this study also show that watching TV, drinking tea with friends or neighbors, religious activities, and taking a walk are the main leisure activities for older people. With regard to the mental health of middle-aged and older people, stress can lead to glycemic excursions. Therefore, participation in relaxing and joyful activities and using reading, listening to music, or gardening as an effective stress management measure can alleviate stress, thereby improving the mental health status of middle-aged and older people. Conversely, participation in leisure activities can provide opportunities to interact with other people, which is vital to the mental and emotional health of middle-aged and older diabetics in the current aged and sub-replacement fertility social environment. This can help alleviate loneliness and depression in patients and in-crease life satisfaction in middle-aged and older people. In middle-aged and older populations with long-term diabetes, these leisure activities can help maintain a positive mindset and face challenges caused by diabetes, thereby improving quality of life. Besides, these four main leisure activities (watching TV, drinking tea with friends or neighbors, religious activities, and taking a walk) are very easy to carry out. Especially for taking a walk, it not only provides opportunities to interact with other people, but also improves the the physical health of older people.

This study used the TLSA that is representative of Taiwan to estimate the effects of leisure activities on healthy life expectancy in middle-aged and older diabetics. This was different from past studies as measurement and observation of the health effects of leisure activities was further extended from purely quantitative calculation (life expectancy) to healthy life expectancy with a qualitative connotation. The results of this study found that participation in leisure activities can decrease the years lived with disability and increase the healthy life expectancy of middle-aged and older people aged 50 years and above. Besides providing evidence that participation in leisure activities can extend life expectancy and maintain health status, the estimation results can also be translated for the development of teaching materials and public communication so that simple life expectancy and healthy life expectancy comparison can be used to help older people understand the positive effects of leisure activities on health. This can also encourage middle-aged and older people to participate in moderate activities to maintain health. On the other hand, we know that comorbidities may also influence life expectancy. However, the comorbidities of the diabetics did not be considered in this study since no information about comorbidities caused by diabetes could be carried out. This is one of limitations of this study. The effect of comorbidities for older people’s life expectancy can treat as a direction for our future study.

This study is a long-term cohort study that is representative of Taiwan and examines the correlation between leisure activities and healthy life expectancy. Its prospective cohort study design helped to elucidate the correlation between leisure activity participation and health status conversion. However, people with better health status may participate in leisure activities, resulting in longer and healthier life expectancy. Overall, participation in leisure activities by middle-aged and older diabetics is a multi-pronged health promotion strategy that can help to comprehensively improve their quality of life, physical health, and mental health. However, patients should consult their physician before starting a new activity plan to ensure the safety and suitability of the activity.

## Data Availability

Data available on request from the authors.
